# Screening of the Hepatotoxic Components in *Fructus Gardeniae* and Their Effects on Rat Liver BRL-3A Cells

**DOI:** 10.3390/molecules24213920

**Published:** 2019-10-30

**Authors:** Chunnan Li, Meng Lan, Jingwei Lv, Ye Zhang, Xiaochen Gao, Xu Gao, Lihua Dong, Guangming Luo, Hui Zhang, Jiaming Sun

**Affiliations:** 1 School of Pharmacy, Jiangxi University of Traditional Chinese Medicine, Nanchang 330004, China; lcn1013@hotmail.com (C.L.); dlh319@126.com (L.D.); 2 Jilin Ginseng Academy, Changchun University of Chinese Medicine, Changchun 130117, China; jingwei-lv@hotmail.com (J.L.); zhangye9584@163.com (Y.Z.); gao_xiaochen@hotmail.com (X.G.); 3 Jilin Xinshui Science and Technology Development Co., Ltd., Changchun 130117, China; lanmengde@163.com (M.L.); jngx_9731@163.com (X.G.)

**Keywords:** *Fructus Gardeniae*, hepatotoxicity, geniposide, genipin, apoptosis, inflammation, BRL-3A rat liver cells

## Abstract

*Fructus Gardeniae* (FG) is a common Chinese medicine and food. However, the toxicity of FG has drawn increasing concern, especially its hepatotoxicity. The purpose of this study was to screen the hepatotoxic components of FG and evaluate their effects on rat liver BRL-3A cells. The chemical composition of FG was determined by HPLC-ESI-MS. CCK-8 assay was used to evaluate the cytotoxicity of ten chemical components from FG, and then the toxic components with significant inhibitory activity were selected for further study. The results showed that geniposide, genipin, genipin-1-gentiobioside, gardenoside, and shanzhiside all suppress cells viability. Apoptosis assays further indicated that geniposide and its metabolite genipin are the main hepatotoxic components of FG. Pretreatment of cells with geniposide or genipin increased the levels of aspartate aminotransferase (AST), alanine aminotransferase (ALT), and alkaline phosphatase (ALP). The activities of superoxide dismutase (SOD) and glutathione (GSH) were decreased, while the malondialdehyde (MDA) level was increased. The cell contents of tumor necrosis factor (TNF-α), interleukin-6 (IL-6), and nitric oxide (NO) were also increased. Molecular docking simulations were used to investigate the mechanism of FG-induced hepatotoxicity, revealing that geniposide and genipin bind strongly to the pro-inflammatory factor TNFR1 receptor of the NF-κB and MAPK signaling pathways. The obtained results strongly indicate that the hepatotoxicity of FG is caused by iridoids compounds. Genipin had the most significant hepatotoxic effect. These toxic substances destroy the cell antioxidant defense system, increasing inflammatory injury to the liver cells and leading to apoptosis and even necrosis. Thus, this study lays a foundation for toxicology research into FG and its rational application.

## 1. Introduction

The worldwide use of traditional Chinese medicine (TCM) in treatments and dietary supplements has increased greatly in recent years. According to a recent survey, 69% of people believe that TCM is mild and has no ill effects [1]. TCMs are regarded as “gentle medicines.” However, further study has revealed that they may cause damage to the liver, kidneys, and other organs [2]. Accordingly, the hepatotoxicity of TCM is attracting increasing attention.

*Gardenia jasminoides* Ellis (Rubiaceae) is a widely used traditional Chinese medicine and health food. It is native to subtropical regions of East Asia, and 250 species are known worldwide. The dried ripe fruit of this plant, known as *Fructus Gardeniae* (FG), is used as a TCM. FG is widely used for the treatment of various conditions, including jaundice, headache, diarrheal, and it has anti-inflammatory and hepatoprotective activity [3,4]. Furthermore, in China, FG is used as a food ingredient and a dietary supplement mixed with tea or healthcare foods.

FG contains iridoids [5,6], crocins [7], and organic acid [8], among others. From reference to the traditional Chinese medicine systems pharmacology database and analysis platform (TCMSP) database, it was found that FG contains 98 chemical components. Two of the major active components in FG are the iridoid glycosides and crocetin derivatives [9]. In recent years, animal studies have shown that extracts of FG cause hepatotoxicity in rats [10,11]. Geniposide is a key bioactive component of FG with levels that vary from 1.8–6%. Biochemical and histopathological examination of rat liver tissues has demonstrated the toxic effects with increasing geniposide dose [12]. Accordingly, increasing numbers of researchers believe that the toxicity of FG is caused by geniposide [13]. However, other scholars believe that the geniposide metabolite genipin and gardenia blue are the main hepatotoxic components of FG [14]. Geniposide forms genipin upon hydrolysis by β-glucosidase in the intestine and is a natural cross linking agent that has high biocompatibility [15]. Gardenia Blue is produced by genipin through amino acid reactions, which demonstrates that it exhibits no genotoxicity toward humans [16]. Thus, the exact components of FG that cause hepatotoxicity are still not clear. FG contains many other chemical components, and basic research into the hepatotoxic components in FG is not extensive and the mechanisms of their action are not known. Evaluating the safety of FG and its major active components is critical for its widespread medical usage.

Therefore, the objective of this study is to screen the components of FG and identify those that exhibit toxic effects in rat liver BRL-3A cells, as well as studying their mechanism of action. It is hoped that this study will provide a scientific basis for the medical development of *Gardenia jasminoides* Ellis.

## 2. Results

### 2.1. Effect of FG Extracton BRL-3A Cells Viability

**Figure 1:**
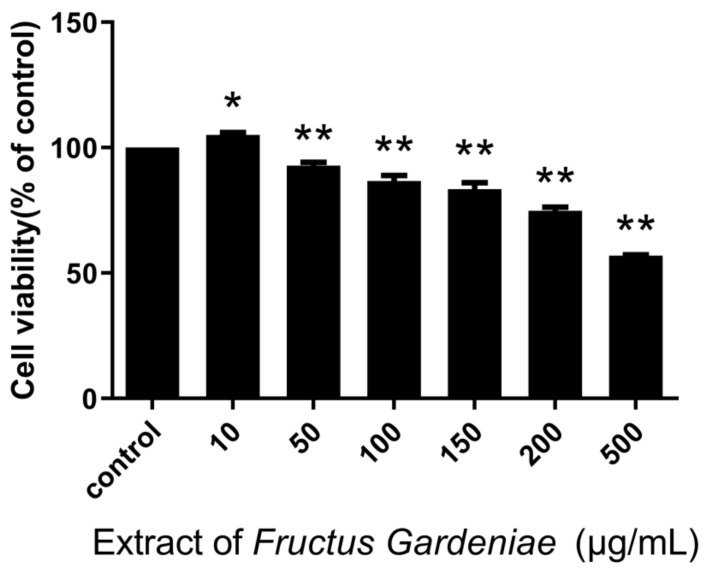
Of Fructus Gardeniae extraction cells viability. Values were presented as mean ± SD, *n* = 6. * *p* < 0.05 and ** *p* < 0.01 vs. control cells.

The CCK8 assay results show that FG extract at concentrations of 50~500 µg/mL inhibits cell proliferation (*p* < 0.05; *p* < 0.01) in a generally dose-dependent manner (Figure 1).

### 2.2. HPLC-ESI-MSAnalysis of FG Extract

**Figure 2:**
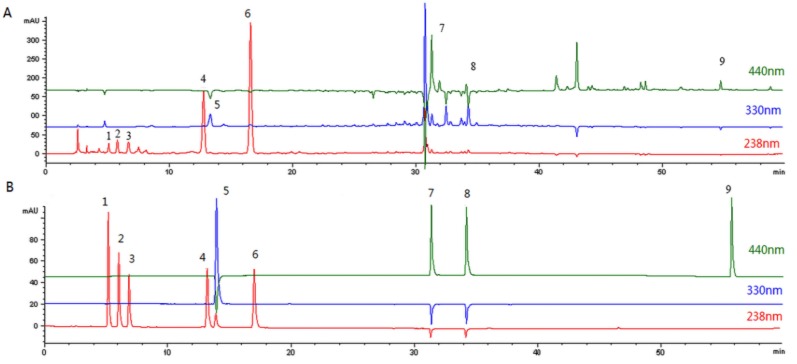
HPLC chromatograms. (**A**) Aqueous extract of *Fructus Gardeniae*. (**B**) Standard chemicals.

The aqueous extract of FG was analyzed by HPLC- ESI-MS and the peaks were compared with those of standard chemicals. Nine compounds were identified by comparing retention times, UV spectra, MS data, and MS^2^ data with literature data [17] (Figure 2). The HPLC- ESI-MS data for the nine peeks are shown in Table 1. For quantitative analysis, calibration curves were prepared using reference materials. All the calibration curves have appropriate linearity. The contents of the nine compounds thus derived are shown in Table 2. The main component of FG is geniposide [18].

### 2.3. Effect of Components on BRL-3A Cells Viability

**Figure 3:**
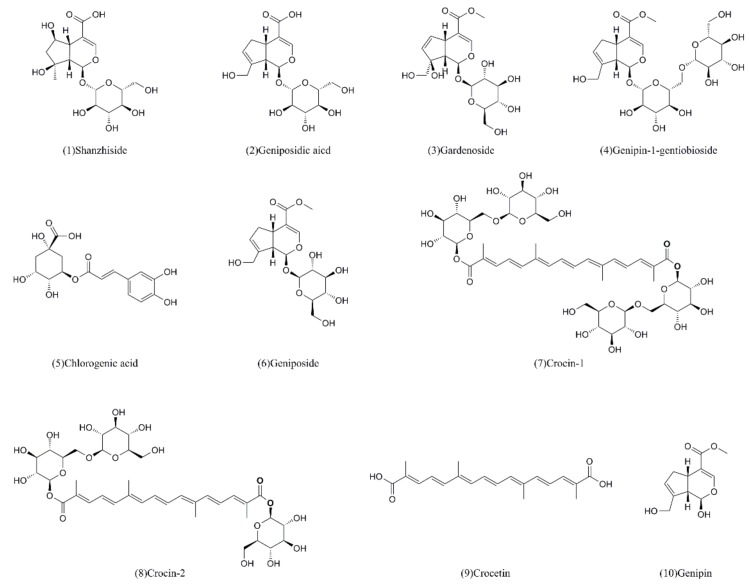
The ten compounds from *Fructus Gardeniae* screened for hepatotoxicity.

The ten standard components (Figure 3) of FG were screened by CCK-8 assay and the resultant cell viabilities were calculated. The results are shown in the Table 3.

The results show that these ten compounds have different effects on cell viability. After treating cells with different concentrations of the compounds, it was found that the crocin-1, crocin-2, crocetin, and normal control group cells appeared to grow well. The cells viabilities in the crocin-1, crocin-2, and crocetin groups(100–500µg/mL) are significantly increased at 24 h compared to the control group (*p* < 0.01). These compounds have the effect of promoting cell proliferation, and their effects decrease in the order crocetin > crocin-2 > crocin-1. The growth of the chlorogenic acid and geniposidic acid group cells are similar to that of the control cells.

Conversely, compared with the control group, the shanzhiside, gardenoside, genipin-1-gentiobioside, geniposide, and genipin groups, exhibit inhibitory effects on cell growth 100–500 µg/mL in a dose-dependent manner (*p* < 0.01). The cell inhibition rates decrease in the order genipin > geniposide > genipin-1-gentiobioside > gardenoside > shanzhiside. These compounds belong to the iridoids family, demonstrating that the hepatotoxicity of FG is mainly derived from such compounds. The EC_50_ values are 154.73 ± 22.6 µg/mL, 358.3 ± 20.78 µg/mL, 1461 ± 10.51 µg/mL, 2743 ± 21.28 µg/mL, and 3005 ± 13.67 µg/mL, respectively.

By polynomial regression analysis, it is found that the curve fitting degree of shanzhiside (R^2^ = 0.872), gardenoside (R^2^ = 0.897), genipin-1-gentiobioside (R^2^ = 0.97), geniposide (R^2^ = 0.978), crocin-1 (R^2^ = 0.99), crocin-2 (R^2^ = 0.97), crocetin (R^2^ = 0.941), and genipin (R^2^ = 0.972) are better and have significant significance. The chlorogenic acid (R^2^ = 0.261) and geniposidic acid (R^2^ = 0.526) factor were not significant.

Thus, FG causes hepatotoxicity not only by geniposide, but by multiple other components. In particular genipin is the most toxic. Therefore, the main component geniposide and the highly toxic genipin were selected for further study.

### 2.4. Effect of Geniposide and Genipin on Aapoptosis and Cell Cycle

**Figure 4:**
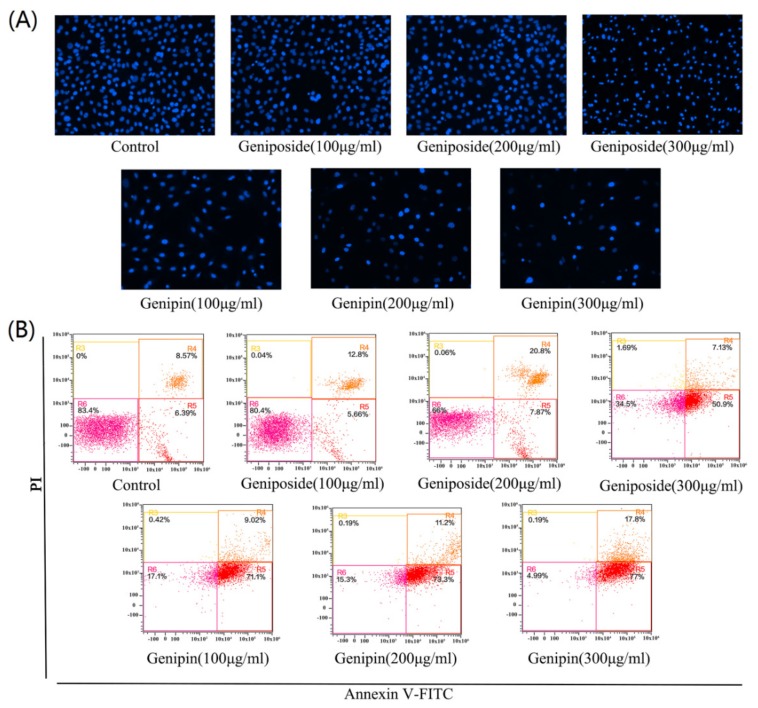
(**A**) DAPI staining results after treatment with geniposide and genipin as photographed with a fluorescence microscope (×200). (**B**) Cell apoptosis as analyzed by flow cytometry.

Figure 4A shows, the staining results or cells treated with geniposide and genipin 100, 200, and 300 µg/mL. With increasing of concentration, the number of cells decreases by cell apoptosis or abscission necrosis. Geniposide causes no significant decrease in cell number at low concentrations (100, and 200 µg/mL), with a significant decrease in cell number only observed at high concentration (300 µg/mL). However, genipin toxicity is very strong, with cells appearing to be necrotic and atrophic and fewer viable cells are observed. With increasing genipin concentration, cell damage becomes more serious and the number of cells decreases significantly.

The results of Annexin V-FITC/PI staining and flow cytometry revealed that the apoptotic rate of the geniposide group increases from 15.6% (control) to 61.65% (300µg/mL); and that of genipin group increases from 15.6%(control) to 91.54% (300µg/mL), as shown in Figure 4B. These results indicate that geniposide and genipin significantly inhibit cell viability and induce apoptosis in cells. In particular, genipin has strong toxic effects, causing almost complete apoptosis of cells at high concentration.

**Figure 5:**
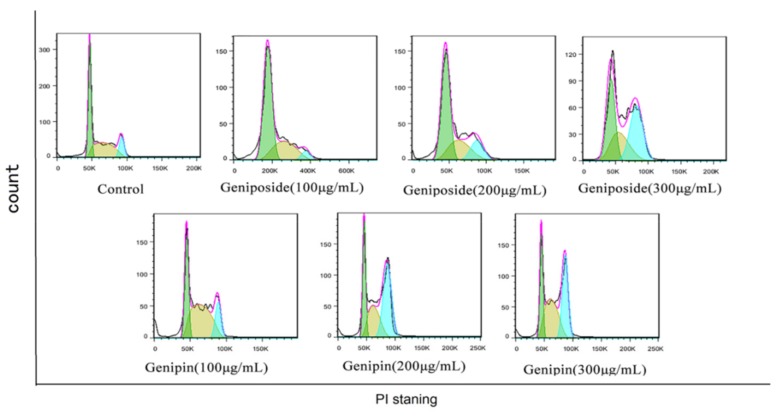
Geniposide and genipin on the BRL-3A cell cycle.

**Figure 6:**
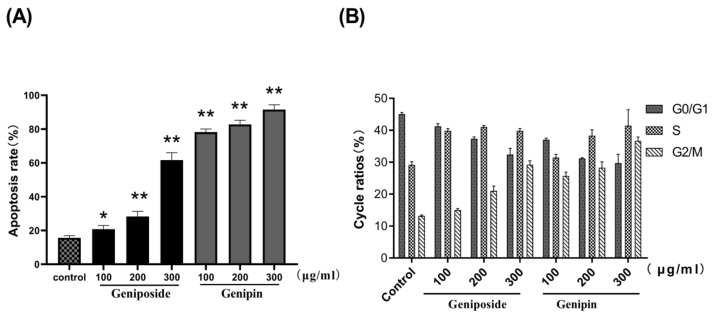
Bar diagrams showing percentage apoptosis and cell cycle. (**A**) Apoptosis diagram. (**B**) Cell cycle diagram. Values are presented as mean ± SD, *n* = 3. * *p* < 0.05 and ** *p* < 0.01 vs. control cells.

The results show that the cell cycles of the geniposide and genipin groups are blocked in the G2/M phase, with the proportion of cells in the S phase increased, and that in the G0/G1 phase decreased compared to those in the control group (Figure 5 and Figure 6).

### 2.5. Effects on Biochemical Indicators, Oxidative Damage Index, and Inflammation

**Figure 7:**
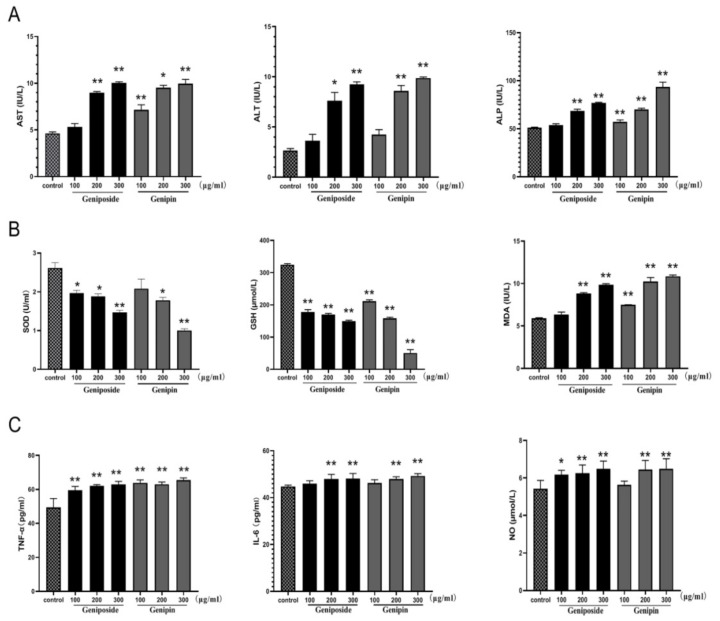
Effects of geniposide and genipin on (**A**) AST, ALT, and ALP; (**B**) SOD, GSH, and MDA; (**C**) TNF-α, IL-6, and NO. BRL3A cells were exposed to geniposide or genipin at 100, 200, and 300µg/mL concentrations. Values are expressed as mean ± SD (*n* = 6), * *p* < 0.05; ** *p* < 0.01 vs. control cells.

As shown in Figure 7A, treatment with geniposide or genipin 100, 200, and 300 µg/mL results in significantly increased aspartate aminotransferase (AST), alanine aminotransferase (ALT), and alkaline phosphatase (ALP) levels in BRL-3A cells compared with those of the control cells (*p* < 0.01). The increased levels of these biochemical indicators indicate that the liver cells have been damaged. The data also show a decrease in superoxide dismutase (SOD) and glutathione (GSH) levels in the geniposide and genipin groups. Furthermore, malondialdehyde (MDA) levels are significantly higher than that of the control group, as shown in Figure 7B. This indicates that they have a significant influence on the oxidative stress of cells.

As shown in Figure 7C, tumor necrosis factor (TNF-α), interleukin-6 (IL-6), and nitric oxide (NO) levels are also markedly increased in the geniposide and genipin groups (200 and 300 µg/mL) as compared to the control group (*p* < 0.01). However, in the low-dose geniposide and genipin groups, only TNF-α is significantly increased, while the inflammatory indicators of IL-6 and NO are not. Thus, under certain doses of geniposide and genipin, cells undergo inflammation.

### 2.6. Molecular Docking

**Figure 8:**
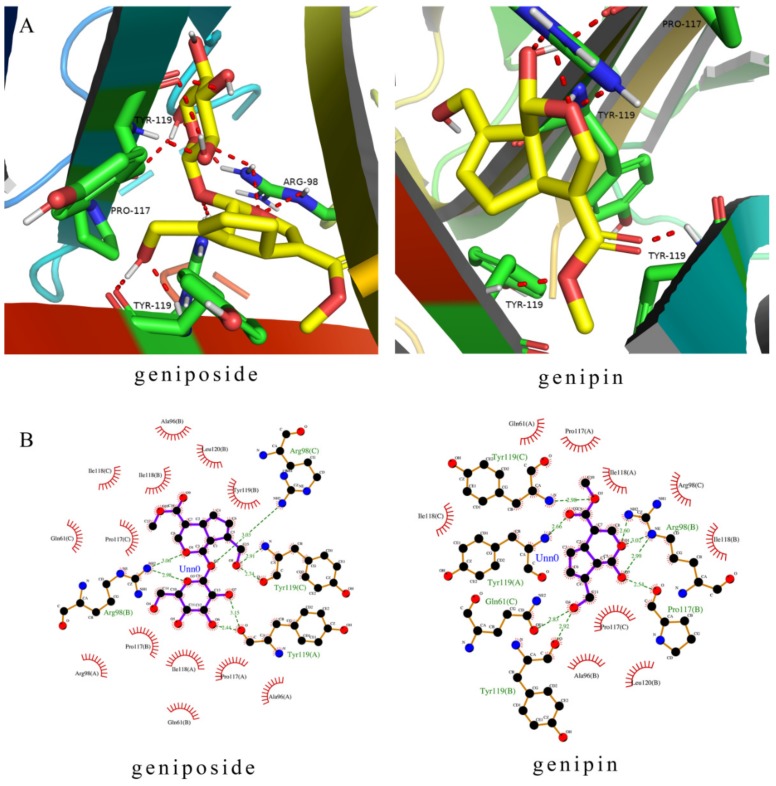
Molecular docking of the geniposide and genipin with TNFR1. (**A**) Molecular ligands are shown in stick form and red dashed lines were hydrogen. (**B**) Two-dimensional (2D) representation of hydrogen bonding and hydrophobic interactions.

Molecular docking revealed the binding modes of the two compounds at the active site in the TNFR1 receptor. Molecular docking simulations reveal how the ligands interact with significant amino acid residues in active sites by hydrogen bonding and hydrophobic interactions. Ligand molecules fall into protein pockets and form interactions with amino acids (Figure 8). The lowest binding energies derived were 7.34 kcal/moL (geniposide) and 6.97 kcal/moL (genipin). The negative binding energy (G < 0) indicated good binding affinity between the compounds and TNFR1. The important residues were shown in Figure 8, including residues in TNFR1 (Arg98, Tyr119, Pro117, and Gln61). Thus the results of molecular docking show that geniposide and genipin bind to the TNFR1 receptor well.

## 3. Discussion

FG is a TCM agent commonly used in clinical applications to treat many conditions and that may also be used for health tea. FG taken at doses recommended by the Chinese Pharmacopoeia (6–10 g/d) generally does not cause organs injury. However, the safety of FG has been questioned, and hepatotoxic analysis of FG has attracted the attention of several researchers. FG hepatotoxicity is generally studied in terms of its aqueous extract, alcoholic extract, and geniposide. Scholars have found that the aqueous and alcoholic extracts exhibit the hepatotoxicity at high doses (≥280 mg/kg) in rats, and the toxicity of the water extract is stronger than that of the alcohol extract [11]. The acute hepatic toxicity of geniposide has been indicated in rats after oral administration at doses of ≥574 mg/kg, and hepatotoxicity often appears at 24–48 h [19]. In addition, HepG2 cells have been previously used to study the hepatotoxicity of geniposide and genipin [14]. However, the extract components are complex, the animals have individual differences, and the HepG2 cells themselves are damaged. Therefore, using standard compounds to directly test normal hepatocytes makes it possible to screen the hepatotoxic components of FG and facilitate analysis of their effects on cells.

Water extraction is a common process by which traditional Chinese medicines are administered. We determined that the extract of FG has inhibitory effect on rat liver BRL-3A cells by the CCK-8 method. Then, we provided a method for the identification of compounds in FG by HPLC-ESI-MS. Through screening the hepatotoxic components of FG, we found that crocins and organic acid do not exhibit hepatotoxicity. In particular, with increasing concentration, the cell activities of the crocin-1, crocin-2, and crocetin groups are improved, which demonstrates that they have hepatoprotective efficacy. This result has been confirmed by animal study [9]. Crocin and crocetin are potent antioxidants and have anti inflammation activity [20,21]. Crocin reduces liver toxicity by protecting mitochondria and lysosomes [22]. Researchers have found that crocin can play a significant role in regulating the PI3K/Akt-mediated inflammatory pathway [23]. Crocetin liver protection may be related to down-regulation of p-p38 MAPK and anti-inflammatory effects [24]. Geniposide has the highest content and is the main bioactive component of FG. However, our results demonstrated that the hepatotoxicity of genipin is greater than those of geniposide and the other components. Genipin may induce apoptosis through biological activity related to the ROS/JNK signaling pathway [25]. In addition, we have demonstrated for the first time that genipin-1-gentiobioside, gardenoside, and shanzhiside also exhibit hepatotoxicity. Genipin-1 -gentiobioside, gardenoside, and shanzhiside have antioxidant effects [26]. Gardenoside may be considered a potential drug candidate that targets P2 × 3 and P2X7 purine receptors [27]. By examining the chemical structures of these toxic substances, we found that they all belong to the iridoid family of compounds. Although their contents in FG are low and easily overlooked, they can be converted to genipin. For example, genipin-1-gentiobioside can undergo removal of a sugar molecule in the intestinal tract to generate geniposide and further generate genipin [28].

In order to further study the toxic effects of FG on hepatocytes, we selected geniposide, the main iridoid component, and genipin, the most toxic component, as the research objects. We demonstrated that geniposide and genipin induce apoptosis in cells. Through cell cycle analysis, we found that geniposide and genipin affect the normal growth cycle of liver cells, with cell arrest occurring in the S and G2 phases similar to that observed for liver cancer cells [29]. In this process, the G2/M transition is regulated by modulating cyclin B1 and cyclin-dependent kinase 1 activities [30].

In the present study, the liver biochemical indicator levels revealed considerable liver cell injury. AST, ALT, and ALP are biomarkers of liver function [31]. ALT is mainly distributed in hepatocyte cytoplasm, and elevated ALT levels indicate damage of hepatocyte membrane. AST is mainly distributed in hepatocyte cytoplasm and hepatocyte mitochondria, and its elevation indicates damage to theses organelles [32,33]. ALT is mainly related to hepatocyte metabolism [34]. Furthermore, we observed that the levels of SOD and GSH are significantly decreased and the level of MDA is increased as compared with those of the control group, demonstrating that the treated liver cells produce a large amount of superoxide and hydroxyl free radicals. SOD and GSH are the major antioxidant enzymes for scavenging free radicals, and most hepatotoxic drugs impair their functions [35]. GSH is considered to be a key antioxidant involved in mitochondrial dysfunction and apoptosis, and a GSH imbalance may lead to redox imbalance [36,37]. MDA is produced by free-radical-mediated lipid peroxidation and is used as a marker of oxidative stress [38,39]. FG promotes the production of free radicals in liver cells, causing an increase in cellular peroxide.

However, inflammation has a pivotal role in hepatotoxicity as the source of oxidative injury. TNF-α and IL-6 are the two major inflammatory cytokines, and they are promptly and transiently produced in response to infection and tissue injury [40,41]. Excessive NO, being an inflammatory mediator can contribute to the liver injury [42]. After geniposide and genipin treatment, the contents of TNF-α, IL-6, and NO in the cell supernatant are increased. These results indicate that with increasing concentration geniposide and genipin may damage liver cells and cause inflammation. Molecular docking further verified the mechanism of this process. TNF is a pro-inflammatory cytokine and the TNFR1 receptor signaling pathway is closely related to many inflammatory diseases [43]. TNFR1 is located upstream of multiple pathways, including NF-κB, MAPK, and TNF signaling pathways [44,45,46]. TNFR1 is activated to present downstream signals, thus activating the NF-κB or MAPK signaling pathways and producing inflammatory reactions. In addition, when TNFR1 binds to a ligand, it can bind to FADD by TRADD and activate caspase-8, and cause apoptosis [47]. The molecular docking study elucidated the binding mode of the two compounds at the active site of TNFR1, with the lowest binding energies indicating better binding. According to the results of molecular docking, FG can activate pro-inflammatory factor TNFR1 and transmit inflammatory signals, thus activating multiple signal pathways. For example, activating the NF-κB pathway key protein p65 will lead to the release of the inflammatory cytokines TNF-α and IL-6, thus causing inflammation. Thus, oxidative stress and inflammation are the main mechanisms of the hepatotoxicity induced by FG.

Through this study, we have identified FG components that contribute to hepatotoxicity. In the future, our research team will further study the signaling pathways, functional proteins, and regulatory genes in vivo that lead to FG-induced hepatotoxicity.

## 4. Materials and Methods

### 4.1. Plant Materials

The dried fruits of G. jasminoides Ellis were collected in October 2017 from Xiashan county, Ji ‘an city, Jiangxi province, China, and purchased from Jiangxi Puzheng Pharm Corp. The plant materials were identified by Prof. Fei Ge (Jiangxi University of Traditional Chinese Medicine, Nanchang, China). The voucher specimens are deposited in school of Pharmacy, Jiangxi University of Traditional Chinese Medicine, Jiangxi, China.

### 4.2. Reagents and Chemicals

Standard compounds geniposide, gardenoside, genipin-1-gentiobioside, shanzhiside, chlorogenicacid, crocin-1, crocin-2, crocetin, geniposidic acid, and genipin with over 98% purity were purchased from Shanghai Yuanye Bio-Technology Co., Ltd. (Shanghai, China). Cell Counting Kit-8 purchased from EnoGene technology Co., Ltd. (Nanjing, China). Annexin V-FITC/PI apoptosis detection kit purchased from BestBio technology Co., Ltd. (Shanghai, China). DAPI staining solution purchased from Beijing Solarbio Science & Technology Co., Ltd. (Beijing, China). TNF-α,IL-6, and NO enzyme-linked immuno sorbent assay (ELISA) kits (R&D, USA) were purchased from Changchun Bestgene Biotechnology Co., Ltd. (Changchun, China). ALT, AST, ALP, MDA, SOD, and GSH kits were obtained from Nanjing JianCheng Institute of Biological Engineering (Nanjing, China). Acetonitrile and Formic acid (Fisher, Fair Lawn, NJ, USA) were of HPLC grade.

### 4.3. Extracts of FG Preparation and HPLC- ESI-MS Analysis

The FG was milled and sieved through a 40-mesh screen before use. Powder of FG was obtained by decocting three times with eight portions of water over 2 h. The decoction is collected and concentrated to dryness under reduced pressure, then used for analysis by HPLC- ESI-MS.

The HPLC was performed on a Agilent 1260 HPLC system quipped with a ZORBAX SB-C18 column (4.6 × 250 mm, 5 μm; Agilent, Palo Alto, CA, USA) kept at 25 °C and at a flow rate of 0.8 mL/min. 0.1% formic acid (A) and acetonitrile (B) were used as mobile phase. The gradient elution of B was performed as follows: 8%–15% B at 0–18 min, 15%–23% B at 18–25 min, 23%–35% B at 25–40 min, 35%–65% B at 40–55 min. The samples injected volume was 10 µl while chromatogram was recorded at 238 nm, 330 nm, and 440 nm.

MSD Trap mass spectrometer 6320 (Agilent, USA) equipped with an electrospray ionization source was used in both positive and negative ion mode. The ESI source parameters were set as follows [48]: gas temperature was 350 °C; capillary voltage was 4000 V; nebulizer pressure was 35 psi; drying gas was 9.0 L/min; skimmer voltage was 60V; sheath gas and auxiliary gas were nitrogen; scan range was 50–1200 *m*/*z*. Comparison the area of chromatographic peaks with the calibration curves enabled quantification of compounds.

### 4.4. Cell Culture

Rat liver cell line BRL-3Awas obtained from the cell bank of the School of Basic Medicine of Beijing Union Medical College (Beijing, China) and was cultured in DMEM medium (GIBCO, Waltham, MA, USA), supplemented with 10% (*v*/*v*) FBS (GIBCO, Australia), penicillin (100 U/mL), and streptomycin (100 U/mL) at 37 °C in an atmosphere containing 5% CO_2_.

### 4.5. Cell Viability Assay

The cell viability was determined by the CCK-8 assay. The cells were seeded into 96-well plates at a density of 4000 cells/well, treated with 10, 50, 100, 150, 200, and 500 µg/mL of samples maintained at 37 °C for 24 h. CCK-8 reagent was added to each well and further incubated for 1 h. Absorbance (OD) was detected at 450 nm in an automatic enzyme labeling apparatus (BIO-RAD-680, Hercules, CA, USA). Each treatment was repeated three times for six experimental wells each. The ten components used to screen hepatotoxicity were all standard compounds. Cell viability (%) was calculated by the equation: (OD of compound-treated cells/OD of control -treated cells) × 100%.

### 4.6. Apoptosis and Cell Cycle Detection

Apoptosis was evaluated by DAPI staining and flow cytometry using an Annexin V-FITC. The cells were cultured in 6-well plates and treated with geniposide and genipin for 12 h. DAPI staining: 10 µl DAPI was added to each well and the cells were incubated at 25 °C for 10 min, washed three times by PBS. The cells were observed by fluorescence microscope. Flow cytometry: cells were harvested and cell suspension was made. A total of 400 µl of cell suspension was suctioned for staining with Annexin V-FITC/PI double staining. The cells were co-incubated with 5 µL of V-FITC and 5 µL of PI for 15 min at room temperature in the dark. Then cells were analyzed by flow cytometry (Amnis, Seattle, WA, USA) within 1 h.

Cell cycle was analyzed by flow cytometry. The cells were harvested and cell suspension was made. The cells were gently fixed with 70% ice wine at −4 °C for 12 h, centrifuged at 3000 rpm for 10 min, and the collected cells were washed with pre-cooled PBS once. Propidium iodide (PI dye solution) was added to ice for 30 min, and then rinsed with PBS.

### 4.7. Determination of the Biochemical Indicator, Oxidative Damage Index, and Inflammation Indicator

BRL-3A cells were cultured in 6-well plates at 3 × 10^5^ cells/well and were exposed to samples for 24 h. The cells supernatant were used to detect TNF-α, IL-6, and NO. Cells fragmentation was carried out by repeated freezing and thawing. ALT, AST, ALP, MDA, SOD, and GSH were assayed using reagent kits according to the instruction manuals [49]. TNF-α, IL-6, and NO were detected by ELISA-Kit.

### 4.8. Molecular Docking

The structure of TNFR1 (PDB ID: 2ZJC, resolution: 2.5 Å) was obtained from the Protein Data Bank (PDBhttp://www.rcsb.org/pdb). The 3D structures of the ligands were mapped and converted using ChemBioDraw Ultra and ChemBio3D Ultra (Cambridge soft Corp., Waltham, MA, USA). Auto dock was used in the molecular docking study to predict the possibility of protein ligands binding to the molecules [50,51]. The grid was then concentrated on the center (40Å, 40Å, 40Å, 0.375Å, central coordinates x = −8.866, y = 25.474, and z = 4.447). The docking results were visualized using PyMOL and LigPlot [52].

### 4.9. Statistical Analysis

All assays are performed at least in triplicate, and the results are expressed as mean ± standard deviation (SD). SPSS software was used for polynomial comparative analysis. The values of EC50 are calculated by GraphPad Prism 8. We statistically assessed the data by using one-way (ANOVA) analysis of variance for post-hoc test (SPSS 22.0). The statistical difference is *p* < 0.05(*), and extreme statistical difference is *p* < 0.01 (**).

## 5. Conclusions

In summary, through identification and hepatotoxic screening of ten compounds from FG, we confirmed that crocins promote cell proliferation, while geniposide and four other compounds inhibit cell growth. Geniposide is the major hepatotoxic component of FG, but it is not the only chemical component that induces hepatotoxicity. Genipin exhibits the strongest toxicity to liver cells and it is speculated that the metabolism of iridoid components structures into genipin is the direct cause of the hepatotoxicity caused by FG. Through studying the effects of these components on cells, it was found that the mechanism of FG-induced hepatotoxicity may be related to destroying the ability of cells to resist oxidative stress, inducing the release of inflammatory cytokines and apoptosis. Therefore, we suggest that FG should not be taken in large doses or for a longer duration. In addition, the discovery of other toxic components in FG will provide new avenues for future FG safety research.

## Figures and Tables

**Table 1:** HPLC- ESI-MS data of nine common peeks.

No.	Retention Time (min)	λmax(nm)	Observed *m*/*z*	Fragment Ion	Component Name
1	5.23	238	414.8 [M + Na]^+^	216.2, 234.3, 252.3	Shanzhiside
2	5.78	238	397.0 [M + Na]^+^	202.0, 216.2, 234.2	Geniposidic acid
3	6.66	238	426.9 [M + Na]^+^	232.2, 246.3, 264.3	Gardenoside
4	13.20	238	573.0 [M + Na]^+^	304.4, 364.5	Genipin-1-gentiobioside
5	13.32	320	376.7 [M + Na]^+^	184.3, 215.3	Chlorogenic acid
6	16.63	238	410.7 [M + Na]^+^	230.2	Geniposide
7	31.28	440	999.7 [M + Na]^+^	346.5, 582.8, 674.8	Crocin-1
8	34.09	440	837.5 [M + Na]^+^	346.5, 512.7, 674.8	Crocin-2
9	54.72	440	350.9 [M + Na]^+^	249.5, 289.5, 306.5	Crocetin

**Table 2:** Equation, determination coefficient, linear range, and compounds content of nine compounds of *Fructus Gardeniae* extract.

Analyte	Regression Equation (Liner Model)	Linear Range(µg/mL)	R^2^	Content (mg/g)
Shanzhiside	y = 14.21x + 18.97	3.8–190	0.9992	2.12 ± 0.11
Geniposidic acid	y = 12.96x + 48.43	4.4–220	0.9994	4.84 ± 0.15
Gardenoside	y = 8.60x + 17.56	3–150	0.9993	4.04 ± 0.02
Genipin-1-gentiobioside	y = 7.28x − 26.28	11.8–590	0.9999	8.41 ± 0.50
Chlorogenic acid	y = 33.06x − 32.16	2.4–120	0.9992	1.14 ± 0.49
Geniposide	y = 11.30x + 51.55	16.2–810	0.9999	46.6 ± 1.14
Crocin-1	y = 18.24x + 15.99	10–500	0.9997	13.9 ± 1.47
Crocin-2	y = 15.24x + 62.03	8–400	0.9996	3.1 ± 0.48
Crocetin	y = 15.07x + 54.47	7.8–390	0.9993	0.81 ± 0.15

R^2^: correlation coefficient of regression equations. Content (mg/g): The compounds content is calculated according to *Fructus Gardeniae* crude drug quantity. Values are expressed as mean ± SD (*n* = 3).

**Table 3:** Compounds of *Fructus Gardeniae* extract on the cell viability of BRL-3A cells (%).

Samples	10 µg/mL	50 µg/mL	100 µg/mL	150 µg/mL	200 µg/mL	500 µg/mL
Shanzhiside	89.14 ± 8.02 **	79.73 ± 4.72 **	75.69 ± 5.23 **	76.53 ± 4.37 **	76.66 ± 4.22 **	63.60 ± 3.58 **
Geniposidic acid	109.71 ± 10.65 **	99.70 ± 1.63	85.03 ± 10.05 **	96.79 ± 2.64	91.77 ± 4.40 *	92.28 ± 2.62 *
Gardenoside	97.59 ± 3.60	95.29 ± 3.36 *	89.02 ± 1.74 **	93.67 ± 0.95 **	85.91 ± 3.38 **	77.39 ± 5.49 **
genipin-1-Gentiobioside	93.22 ± 9.63	92.93 ± 13.15 *	76.43 ± 9.79 **	89.81 ± 8.74 *	82.46 ± 10.18 **	64.62 ± 8.51 **
Chlorogenic acid	95.21 ± 3.30 *	86.84 ± 4.91 **	94.10 ± 0.86 *	89.53 ± 3.67 *	100.50 ± 0.45	98.21 ± 2.03
Geniposide	98.88 ± 2.39	81.38 ± 2.27 **	72.78 ± 3.09 **	67.93 ± 1.51 **	57.81 ± 1.62 **	43.15 ± 0.97 **
Crocin-1	85.29 ± 8.46 **	105.57 ± 4.05	110.25 ± 4.87 **	126.32 ± 9.93 **	142.79 ± 6.81 **	186.74 ± 4.57 **
Crocin-2	117.25 ± 7.61 *	126.52 ± 15.73 **	144.66 ± 7.48 **	141.21 ± 8.66 **	161.16 ± 17.77 **	191.43 ± 10.33 **
Crocetin	153.53 ± 19.22 **	153.18 ± 19.95 **	163.79 ± 18.09 **	196.94 ± 29.78 **	235.18 ± 35.72 **	327.49 ± 26.22 **
Genipin	86.16 ± 1.73 **	77.80 ± 7.22 **	53.76 ± 2.25 **	42.99 ± 5.14 **	41.76 ± 1.48 **	38.98 ± 2.61 **

Values are expressed as mean ± SD (*n* = 6); * *p* < 0.05 and ** *p* < 0.01 vs. control cell (100%).

## References

[1] Chow H.C., So T.H., Choi H.C.W., Lam K.O. (2019). Literature Review of Traditional Chinese Medicine Herbs-Induced Liver Injury from an Oncological Perspective with RUCAM. Integr. Cancer Ther..

[2] Efferth T., Kaina B. (2011). Toxicities by herbal medicines with emphasis to traditional Chinese medicine. Curr. Drug Metab..

[3] Zongram O., Ruangrungsi N., Palanuvej C., Rungsihirunrat K. (2017). Standardization of Gardenia jasminoides Fruits and Crocin Content Analysis Using UV/visible Spectrophotometry. Chiang Mai J. Sci..

[4] Yokoyama Y., Nagino M. (2014). Current scenario for the hepatoprotective effects of Inchinkoto, a traditional herbal medicine, and its clinical application in liver surgery: A review. Hepatol. Res..

[5] Yu Y., Xie Z.L., Gao H., Ma W.W., Dai Y., Wang Y., Zhong Y., Yao X.S. (2009). Bioactive iridoid glucosides from the fruit of Gardenia jasminoides. J. Nat. Prod..

[6] Li L., Zou J., Xia Q., Cui H., You S., Liu Y., Wang Q. (2018). Anti-TMV and Insecticidal Potential of Four Iridoid Glycosides from Gardenia Jasminoides Fruit. Chem. Res. Chin. Univ..

[7] Ni Y., Li L., Zhang W., Lu D., Zang C., Zhang D., Yu Y., Yao X. (2017). Discovery and LC-MS Characterization of New Crocins in Gardeniae Fructus and Their Neuroprotective Potential. J. Agric. Food Chem..

[8] Kim H.J., Kim E.J., Seo S.H., Shin C.G., Jin C., Lee Y.S. (2006). Vanillic acid glycoside and quinic acid derivatives from Gardeniae Fructus. J. Nat. Prod..

[9] Chen P., Chen Y., Wang Y., Cai S., Deng L., Liu J., Zhang H. (2016). Comparative Evaluation of Hepatoprotective Activities of Geniposide, Crocins and Crocetin by CCl4-Induced liver Injury in Mice. Biomol. Ther..

[10] Cui Y.Z., Sun R., Wang Q.J., Wang M.Z. (2017). Hepatotoxicity induced by intragastrically administrated with Gardenia decoction in mice. Nat. Prod. Res..

[11] Yang H.J., Fu M.H., Wu Z.L., Liang R.X., Huang L.Q., Fang J., Li G., Cao Y. (2006). Experimental studies on hepatotoxicity of rats induced by Fructus Gardeniae. Zhongguo Zhong Yao Za Zhi Zhongguo Zhongyao Zazhi China J. Chin. Mater. Med..

[12] Wang Y., Feng F. (2019). Evaluation of the Hepatotoxicity of the Zhi-Zi-Hou-Po Decoction by Combining UPLC-Q-Exactive-MS-Based Metabolomics and HPLC-MS/MS-Based Geniposide Tissue Distribution. Molecules.

[13] Tian J., Yi Y., Zhao Y., Li C., Zhang Y., Wang L., Pan C., Han J., Li G., Li X. (2018). Oral chronic toxicity study of geniposide in rats. J. Ethnopharmacol..

[14] Khanal T., Kim H.G., Choi J.H., Do M.T., Kong M.J., Kang M.J., Noh K., Yeo H.K., Ahn Y.T., Kang W. (2012). Biotransformation of geniposide by human intestinal microflora on cytotoxicity against HepG2 cells. Toxicol. Lett..

[15] Sung H.W., Huang R.N., Huang L.L., Tsai C.C., Chiu C.T. (1998). Feasibility study of a natural crosslinking reagent for biological tissue fixation. J. Biomed. Mater. Res..

[16] Hobbs C.A., Koyanagi M., Swartz C., Davis J., Maronpot R., Recio L., Hayashi S.M. (2018). Genotoxicity evaluation of the naturally-derived food colorant, gardenia blue, and its precursor, genipin. Food Chem. Toxicol..

[17] Han Y., Wen J., Zhou T., Fan G. (2015). Chemical fingerprinting of Gardenia jasminoides Ellis by HPLC-DAD-ESI-MS combined with chemometrics methods. Food Chem..

[18] Shi F., Pan H., Li Y., Huang L., Wu Q., Lu Y. (2018). A sensitive LC-MS/MS method for simultaneous quantification of geniposide and its active metabolite genipin in rat plasma and its application to a pharmacokinetic study. Biomed. Chromatogr..

[19] Ding Y., Zhang T., Tao J.S., Zhang L.Y., Shi J.R., Ji G. (2013). Potential hepatotoxicity of geniposide, the major iridoid glycoside in dried ripe fruits of Gardenia jasminoides (Zhi-zi). Nat. Prod. Res..

[20] Alavizadeh S.H., Hosseinzadeh H. (2014). Bioactivity assessment and toxicity of crocin: A comprehensive review. Food Chem. Toxicol..

[21] Hsu J.D., Chou F.P., Lee M.J., Chiang H.C., Lin Y.L., Shiow S.J., Wang C.J. (1999). Suppression of the TPA-induced expression of nuclear-protooncogenes in mouse epidermis by crocetin via antioxidant activity. Anticancer Res..

[22] Yousefsani B.S., Mehri S., Pourahmad J., Hosseinzadeh H. (2018). Crocin Prevents Sub-Cellular Organelle Damage, Proteolysis and Apoptosis in Rat Hepatocytes: A Justification for Its Hepatoprotection. Iran. J. Pharm. Res. IJPR.

[23] Xie Y., He Q., Chen H., Lin Z., Xu Y., Yang C. (2019). Crocin ameliorates chronic obstructive pulmonary disease-induced depression via PI3K/Akt mediated suppression of inflammation. Eur. J. Pharm..

[24] Diao S.L., Sun J.W., Ma B.X., Li X.M., Wang D. (2018). Influence of crocetin on high-cholesterol diet induced atherosclerosis in rats via anti-oxidant activity together with inhibition of inflammatory response and p38 MAPK signaling pathway. Saudi J. Biol. Sci..

[25] Kang M.J., Khanal T., Kim H.G., Lee D.H., Yeo H.K., Lee Y.S., Ahn Y.T., Kim D.H., Jeong H.G., Jeong T.C. (2012). Role of metabolism by human intestinal microflora in geniposide-induced toxicity in HepG2 cells. Arch. Pharmacal. Res..

[26] Shan M.Q., Wang T.J., Jiang Y.L., Yu S., Yan H., Zhang L., Wu Q.N., Geng T., Huang W.Z., Wang Z.Z. (2019). Comparative analysis of sixteen active compounds and antioxidant and anti-influenza properties of Gardenia jasminoides fruits at different times and application to the determination of the appropriate harvest period with hierarchical cluster analysis. J. Ethnopharmacol..

[27] Yu M., Zhao Y., Zhang X. (2018). Gardenoside combined with ozone inhibits the expression of P2 × 3 and P2 × 7 purine receptors in rats with sciatic nerve injury. Mol. Med. Rep..

[28] Jiang P., Ma Y., Gao Y., Li Z., Lian S., Xu Z., Jiang W., Tian X., Huang C. (2016). Comprehensive Evaluation of the Metabolism of Genipin-1-beta-d-gentiobioside in Vitro and in Vivo by Using HPLC-Q-TOF. J. Agric. Food Chem..

[29] Liu L.L., Zhu J.M., Yu X.N., Zhu H.R., Shi X., Bilegsaikhan E., Guo H.Y., Wu J., Shen X.Z. (2019). UBE2T promotes proliferation via G2/M checkpoint in hepatocellular carcinoma. Cancer Manag. Res..

[30] Park C., Cha H.J., Choi E.O., Lee H., Hwang-Bo H., Ji S.Y., Kim M.Y., Kim S.Y., Hong S.H., Cheong J. (2019). Isorhamnetin Induces Cell Cycle Arrest and Apoptosis Via Reactive Oxygen Species-Mediated AMP-Activated Protein Kinase Signaling Pathway Activation in Human Bladder Cancer Cells. Cancers.

[31] Kamel E.O., Hassanein E.H.M., Ahmed M.A., Ali F.E.M. (2019). Perindopril ameliorates hepatic IR injury via regulation of NF-kappaB-p65/TLR-4, JAK1/STAT-3, Nrf-2 and PI3K/Akt/mTOR signaling pathways. Anat. Rec..

[32] Abdel-Gaber S.A., Geddawy A., Moussa R.A. (2019). The hepatoprotective effect of sitagliptin against hepatic ischemia reperfusion-induced injury in rats involves Nrf-2/HO-1 pathway. Pharmacol. Rep. Pr..

[33] Yang J., Zhu A., Xiao S., Zhang T., Wang L., Wang Q., Han L. (2019). Anthraquinones in the aqueous extract of Cassiae semen cause liver injury in rats through lipid metabolism disorder. Phytomed. Int. J. Phytother. Phytopharm..

[34] Sharma U., Pal D., Prasad R. (2014). Alkaline phosphatase: An overview. Indian J. Clin. Biochem. IJCB.

[35] Sanjeev S., Bidanchi R.M., Murthy M.K., Gurusubramanian G., Roy V.K. (2019). Influence of ferulic acid consumption in ameliorating the cadmium-induced liver and renal oxidative damage in rats. Environ. Sci. Pollut. Res. Int..

[36] Franco R., Cidlowski J.A. (2012). Glutathione Efflux and Cell Death. Antioxid. Redox Signal..

[37] Chen D., Zhang X.Y., Sun J., Cong Q.J., Chen W.X., Ahsan H.M., Gao J., Qian J.J. (2019). Asiatic Acid Protects Dopaminergic Neurons from Neuroinflammation by Suppressing Mitochondrial Ros Production. Biomol. Ther..

[38] Zhao L., Jiang Y., Ni Y.X., Zhang T.Z., Duan C.C., Huang C., Zhao Y.J., Gao L., Li S.Y. (2017). Protective effects of Lactobacillus plantarum C88 on chronic ethanol-induced liver injury in mice. J. Funct. Foods.

[39] Wang Y.Z., Li Y.X., Xie J.M., Zhang Y., Wang J.L., Sun X.L., Zhang H.P. (2013). Protective effects of probiotic Lactobacillus casei Zhang against endotoxin- and D-galactosamine-induced liver injury in rats via anti-oxidative and anti-inflammatory capacities. Int. Immunopharmacol..

[40] Tanaka T., Narazaki M., Kishimoto T. (2014). IL-6 in inflammation, immunity, and disease. Cold Spring Harb. Perspect. Biol..

[41] Zhang D., Tang J., Zhang J., Zhang L., Hu C.X. (2019). Responses of pro- and anti-inflammatory cytokines in zebrafish liver exposed to sublethal doses of Aphanizomenon flosaquae DC-1 aphantoxins. Aquat. Toxicol..

[42] Huang W., Wang Y., Jiang X., Sun Y., Zhao Z., Li S. (2017). Protective Effect of Flavonoids from Ziziphus jujuba cv. Jinsixiaozao against Acetaminophen-Induced Liver Injury by Inhibiting Oxidative Stress and Inflammation in Mice. Molecules.

[43] Aggarwal B.B. (2003). Signalling pathways of the TNF superfamily: A double-edged sword. Nat. Rev. Immunol..

[44] Zheng Y., Li S., Hu R., Cheng F., Zhang L. (2019). GFI-1 Protects Against Lipopolysaccharide-Induced Inflammatory Responses and Apoptosis by Inhibition of the NF-kappaB/TNF-alpha Pathway in H9c2 Cells. Inflammation.

[45] Weiler J., Dittmar T. (2019). Minocycline impairs TNF-alpha-induced cell fusion of M13SV1-Cre cells with MDA-MB-435-pFDR1 cells by suppressing NF-kappaB transcriptional activity and its induction of target-gene expression of fusion-relevant factors. Cell Commun. Signal. Ccs.

[46] Dyari H.R.E., Rawling T., Chen Y., Sudarmana W., Bourget K., Dwyer J.M., Allison S.E., Murray M. (2017). A novel synthetic analogue of omega-3 17,18-epoxyeicosatetraenoic acid activates TNF receptor-1/ASK1/JNK signaling to promote apoptosis in human breast cancer cells. Faseb J. Off. Publ. Fed. Am. Soc. Exp. Biol..

[47] Jiang Y., Yu M., Hu X., Han L., Yang K., Ba H., Zhang Z., Yin B., Yang X.P., Li Z. (2017). STAT1 mediates transmembrane TNF-alpha-induced formation of death-inducing signaling complex and apoptotic signaling via TNFR1. Cell Death Differ..

[48] Gao X.C., Lv J.W., Li C.N., Zhang N.X., Tian L.L., Han X.Y., Zhang H., Sun J.M. (2019). Screening of the Active Component Promoting Leydig Cell Proliferation from Lepidium meyenii Using HPLC-ESI-MS/MS Coupled with Multivariate Statistical Analysis. Molecules.

[49] Zhang H.Y., Liu H., Yang M., Wei S.F. (2012). Antithrombotic activities of aqueous extract from Gardenia jasminoides and its main constituent. Pharm Biol..

[50] Sanner M.F. (1999). Python: A programming language for software integration and development. J. Mol. Graph. Model..

[51] Patil M., Noonikara-Poyil A., Joshi S.D., Patil S.A., Patil S.A., Bugarin A. (2019). New Urea Derivatives as Potential Antimicrobial Agents: Synthesis, Biological Evaluation, and Molecular Docking Studies. Antibiotics.

[52] Laskowski R.A., Swindells M.B. (2011). LigPlot+: Multiple ligand-protein interaction diagrams for drug discovery. J. Chem. Inf. Modeling.

